# Acute myocarditis following Comirnaty vaccination in a healthy man with previous SARS-CoV-2 infection

**DOI:** 10.1016/j.radcr.2021.07.082

**Published:** 2021-08-02

**Authors:** Anna Patrignani, Nicolò Schicchi, Francesca Calcagnoli, Elena Falchetti, Nino Ciampani, Giulio Argalia, Antonio Mariani

**Affiliations:** a Cardiology Department, Senigallia Hospital, ASUR Area Vasta 2, Via Cellini n°1, 60019, Senigallia (AN), Marche, Italy; b Radiology Department, Azienda Ospedaliero-Universitaria Ospedali Riuniti di Ancona, Marche, Italy

**Keywords:** Covid-19, Vaccine, BNT162b2, Myocarditis, Adverse reaction

## Abstract

Myopericarditis following mRNA Covid-19 vaccination has recently been reported to health authorities in a lot of countries. They can occur in very rare cases after either the Moderna (mRNA-1273 - Spikevax) or Pfizer-BioNTech (BNT162b2 - Comirnaty) vaccination. Cases predominately occur in younger adult men within 14 days following the second dose. In this article, we present a 56 year-old man with no prior medical history, whit the exception of a mild Covid-19 infection 4 months earlier, who experienced an episode of acute epigastric pain, profuse sweating, tachycardia, hypotension 4 days after the first dose of BNT162b2 vaccine. Troponin I level was elevated. Chest X-ray, electrocardiogram, echocardiogram, coronary angiography didn't show significant abnormalities. Cardiac Magnetic Resonance showed a pattern of acute myocarditis. The condition appeared to be self-limited and the patient recovered without specific therapy. No report of acute myocarditis was observed in the BNT162b2 and mRNA-1273 trials and very rare cases, in comparison to given doses, have been reported to pharmacovigilance systems worldwide. Further surveillance and evaluation of this side effect are warranted to establish the correct balance of benefits and risks of Covid-19 mRNA vaccines, above all in children and younger people (categories with the higher reactogenicity and the lower risk of Covid-19 complications). At the present time the benefits of Covid-19 vaccination significantly exceed possible risks.

## Introduction

Myopericarditis following mRNA Covid-19 vaccination has recently been reported to health authorities in a lot of countries. Cases predominately occur in younger adult men within 14 days following the second dose of either the Moderna (mRNA-1273 - Spikevax) or Pfizer-BioNTech (BNT162b2 - Comirnaty) vaccines. On July 9, 2021 the European Medicine Agency (EMA)’s Safety Committee has concluded that myocarditis and pericarditis can occur in very rare cases following vaccination with Covid-19 mRNA vaccines. The Committee is therefore recommending listing myocarditis and pericarditis as new side effects in the product information for these vaccines, together with a warning to raise awareness among healthcare professionals and people taking these vaccines [1]. In reaching its conclusion, the Committee took into consideration all currently available evidence. This included an in-depth review of 145 cases of myocarditis and 138 cases of pericarditis in the European Economic Area (EEA) among people who received either the Comirnaty or the Spikevax vaccine. As of 31 May 2021, around 177 million doses of Comirnaty and 20 million doses of Spikevax had been given in the EEA. In addition, the Committee also looked into cases received worldwide.

## Case report description

The present report describes a case of acute myocarditis developed within 4 days of the first dose of BNT162b2 mRNA vaccine, in a patient with previous SARS-CoV-2 mild infection.

An otherwise healthy 56 year-old man presented to the emergency department describing the following clinical symptoms, lasted 4 hours and spontaneously regressed on arrival at the hospital: in the middle of the night, acute onset of epigastric pain, profuse sweating, tachycardia, hypotension. Four days before he received the first dose of BNT162b2 mRNA Covid-19 vaccine. He did not report fever, systemic symptoms or cutaneous rash. Four months earlier, he experienced mild signs of Covid-19 infection with fever lasting for 3 days, but he did not complain of chest pain or dyspnea.

**Fig. 1:**
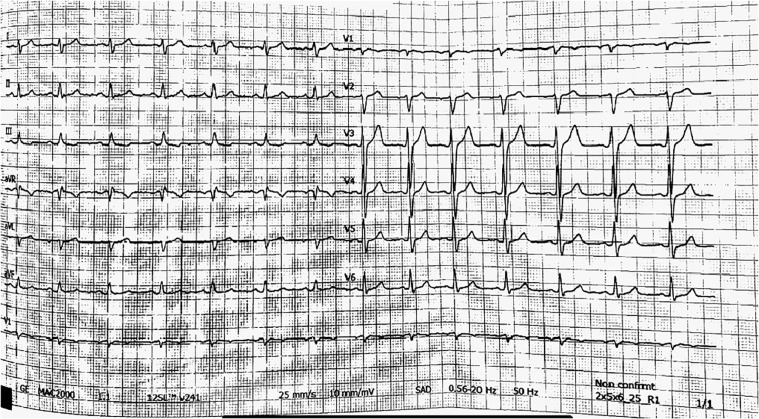
ECG recording on arrival at the emergency department, showing sinus rhythm and no significant abnormalities.

**Fig. 2:**
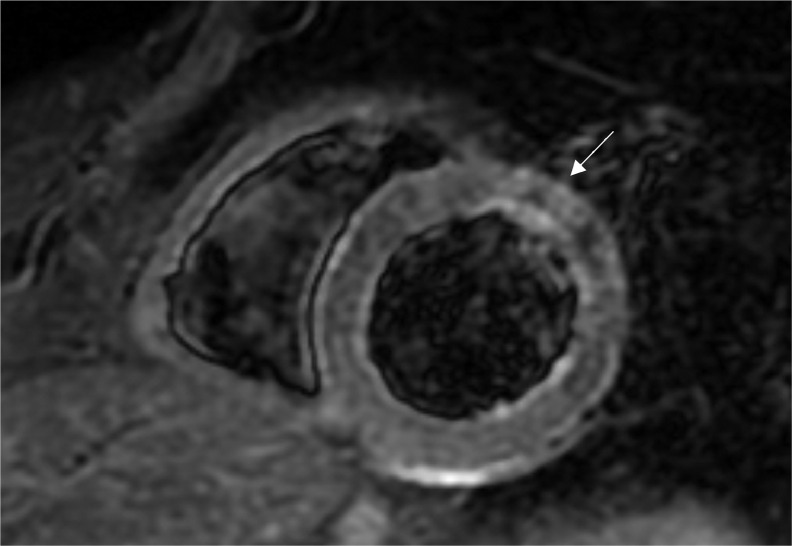
Cardiac magnetic resonance T2-weighted short axis view, showing focal area of edema involving the intramyocardial region of the middle segment of the anterior wall (white arrow).

**Fig. 3:**
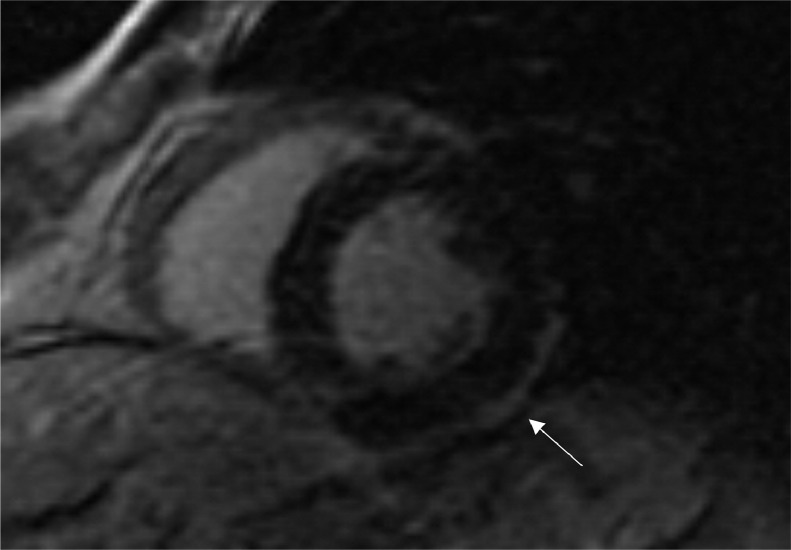
Cardiac magnetic resonance late gadolinium enhancement (LGE) short axis view, showing the presence of sub-epicardial (non-ischemic) lesion in the middle segment of the infero-lateral wall (white arrow).

On arrival at the emergency department arterial blood pressure was 115/80 mmHg, heart rate 86 beats per minute, oxygen saturation 99% while breathing ambient air and body temperature 36°C. Electrocardiogram (ECG) (Fig. 1), chest x-ray and echocardiogram didn't show significant abnormalities. Laboratory tests revealed elevated levels of high-sensitivity (hs) Troponin I (biomarker of myocardial damage) pick 254 ng/L, normal levels of D-dimer, C-reactive protein, white cell count. SARS-CoV-2 nasopharyngeal swab by real-time reverse-transcriptase–polymerase-chain-reaction (rRT-PCR) resulted negative. Coronary angiography, performed 2 days later, didn't show significant narrowing of the coronary arteries. No specific anti-inflammatory or steroidal therapy was administered, and hs-Troponin I levels normalized within 3 days. A Cardiac Magnetic Resonance was performed 7 days later and showed non-dilated ventricles with preserved left (67%) and right ejection fraction (60%). On T2 weighted images, there were focal areas of edema involving the intramyocardial regions of the anterior wall and of the basal and middle segments of the infero-lateral wall (Fig. 2). Late gadolinium enhancement (LGE) confirmed the presence of sub-epicardial (non-ischemic) lesions in the basal and middle segments of the infero-lateral wall, consistent with acute myocarditis (Fig. 3). No pericardial effusion was observed. During hospital stay, no further episodes of chest pain and no arrhythmias were observed on ECG monitoring. After 1 month of follow-up, the patient was asymptomatic and no ECG or echocardiographic abnormalities were evident.

## Discussion

Covid-19 caused by SARS-CoV-2 infection has been demonstrated to be associated with cardiac injury [2]. Cases of acute myocarditis have been reported, even in patients with Covid-19 in the absence of significant lung involvement, suggesting a viral triggered immune-mediated injury [3]. The mRNA vaccines, that encode the prefusion SARS-CoV-2 spike glycoprotein, have shown to confer a very high protection against Covid-19 with a safe profile [4], [5]. On May 10, 2021, the Food and Drug Administration issued an Emergency Use Authorization (EUA) for the Pfizer-BioNTech Covid-19 mRNA vaccine for prevention of Covid-19 for children 12 years and older [6]. Although these vaccines can counteract the Covid-19 pandemic, there is apprehension for “systemic reactogenicity” and especially for myocarditis or myopericarditis. Reactogenicity refers to a subset of reactions that occur soon after vaccination and are a physical manifestation of the inflammatory response to vaccination [7].

Post-immunization myocarditis is a known rare adverse event following other vaccinations, particularly following smallpox vaccination [8] or influenza vaccination [9]. Vaccine-associated autoimmunity is a well-known phenomenon attributed to either the cross-reactivity between antigens or the effect of adjuvants [10]. When coming to Covid-19 vaccines, this matter is further complicated by the nucleic acid formulation and the accelerated development process imposed by the emergency pandemic situation [11]. Although the results from clinical trials have not raised serious safety concerns [4], [5], it is possible that uncommon adverse reactions to Covid-19 vaccines may have not been captured due to low rate of myocarditis and the relatively limited size of the population that received these vaccines before their authorization. Reported local and systemic adverse events seemed to be dose-dependent and more common in participants aged under 55 years. These results presumably depend on the higher reactogenicity occurring in younger people, that may confer greater protection towards viral antigens but also predispose to a higher burden of immunological side effects [12].

Since the beginning of the Covid-19 vaccination campaign cases of acute myocarditis after mRNA vaccines have been described. An Israeli article describes 6 cases of acute myocarditis in males with a median age of 23 years, 5 of them presented 24 to 72 hour after receiving the second dose of the BNT162b2 vaccine and 1 of them presented 16 days after receiving the first dose of the vaccine [13]. A prepublication release on Pediatrics, the official Journal of the American Academy of Pediatrics, describes 7 cases of clinical myocarditis or myopericarditis that developed in the United States in 14 to 19- year-old males, within 4 days of receiving the second dose of the BNT162b2 vaccine, with no history of previous SARS-CoV-2 infection or evidence of acute SARS-CoV-2 infection [14]. An Italian case report describes acute myocarditis after 3 days of the second dose of the BNT162b2 vaccine in a 56 year-old man with previous Covid-19 [15]. A Spanish case report describes acute myocarditis in a 39 year-old man with no history of Covid-19, few hours post-vaccination with the second dose of the BNT162b2 vaccine [16]. An American case report describes acute myocarditis in a 24 year-old man who presented to the hospital with acute substernal chest pain, 4 days after his second dose of mRNA-1273 vaccine [17]. Another American case report describes acute myocarditis in a 20 year-old male who received the BNT162b2 vaccine 2 days before; he had a previous infection with SARS-CoV-2 approximately 2 months earlier [18]. A recent paper published online on June 29, 2021 describes 4 cases of acute myocarditis occurred within 5 days of Covid-19 m-RNA vaccination (3 young males and one 70 year-old female) [19]. Always on June 29, 2021 a retrospective case series study has been published online; it describes patients within the US Military Health System who experienced myocarditis after Covid-19 vaccination between January and April 2021. A total of 23 male patients (median age 25 years) presented with acute onset of marked chest pain within 4 days after receipt of an mRNA vaccine. All military members were previously healthy with a high level of fitness. Seven received the BNT162b2-mRNA vaccine and 16 received the mRNA-1273 vaccine [20].

This case report adds itself to the list presented above and highlights a “potential” relationship between the mRNA BNT162b2 vaccine and cardiac involvement, compatible with acute myocarditis, in an otherwise healthy patient, with a previous mild SARS-CoV-2 infection.

No report of acute myocarditis was observed in the BNT162b2 and mRNA-1273 trials and very rare cases, in comparison to given doses, have been reported to national pharmacovigilance systems worldwide. However, this side effect can be underestimated for several reasons. Clinical trials enrolled not enough people to observe this rare adverse event. A mild form of acute myocarditis can determine non-specific symptoms that don't lead the subject to require medical assistance. Another important reason is the under-use of Cardiovascular Magnetic Resonance in myocardial infarction with non-obstructive coronary arteries (MINOCA) patients [21]. The under-reporting phenomena, well known and well described in literature [22], [23], can contribute to the underestimation of this adverse event in the post-marketing surveillance phase.

Available data suggest that the course of myocarditis and pericarditis following vaccination is similar to the typical course of these conditions. The clinical presentation is highly variable ranging from subclinical disease to acute or slowly progressing heart failure [24], arrhythmias [25] or sudden cardiac death [26]. The clinical course of the majority of published cases was mild; in 5 cases that occurred in the EEA, people died [1]; actually, long-term consequences of these forms of myocarditis are unknown.

Further surveillance and evaluation of these adverse events is warranted to establish the correct balance of benefits and risks of Covid-19 mRNA vaccines above all in children and young people (categories with the higher reactogenicity and the lower risk of Covid-19 complications). Myocarditis should be “actively searched in clinical trials” through routine pre and post vaccination Troponin level measurements, to verify its prospective incidence. This kind of research, for example, demonstrated that passive surveillance significantly underestimated the true incidence of myocarditis and/or pericarditis after smallpox immunization [27]. “Clinical trial registries” should be created to collect long-term data about cases of mRNA vaccine-related myopericarditis.

At the present time the benefits of Covid-19 vaccination significantly exceed possible risks. Individuals and physicians are encouraged to follow the guidance of national health systems on immunization practices. It's of pivotal importance that all cases of myocarditis with or without pericarditis occurring after Covid-19 vaccination should be promptly reported to national pharmacovigilance systems.

## Patient Consent

Informed consent has been provided by the patient.

## References

[1] European Medicines Agency. Comirnaty and Spikevax: possible link to very rare cases of myocarditis and pericarditis. News 09/07/2021. Available at: https://www.ema.europa.eu/en/news/comirnaty-spikevax-possible-link-very-rare-cases-myocarditis-pericarditis.

[2] Shi S, Qin M, Shen B, Cai Y, Liu T (2020). Association of Cardiac Injury With Mortality in Hospitalized Patients With COVID-19 in Wuhan, China. JAMA Cardiol.

[3] Inciardi RM, Lupi L, Zaccone G, Italia L, Raffo M (2020). Cardiac involvement in a patient with coronavirus disease 2019 (COVID-19). JAMA Cardiol.

[4] Polack FP, Thomas SJ, Kitchin N, Absalon J, Gurtman A (2020). Safety and efficacy of the BNT162b2 mRNA Covid-19 vaccine. N Engl J Med.

[5] Baden LR, El Sahly HM, Essink B, Kotloff K, Frey S (2021). Efficacy and safety of the mRNA-1273 SARS-CoV-2 vaccine. N Engl J Med.

[6] Pfizer-BioNTech. Full Emergency Use Authorization (EUA) Prescribing Information, Revised 2021. Accessed July 10, 2021. Available at: http://labeling.pfizer.com/ShowLabeling.aspx?id=14471&format=pdf&#page=13.

[7] Hervé C., Laupèze B., Del Giudice G., Didierlaurent AM, Tavares F (2019). The how’s and what’s of vaccine reactogenicity. npj Vaccines.

[8] Halsell JS, Riddle JR, Atwood E, Gardner P, Shope R (2003). Myopericarditis following smallpox vaccination among vaccinia-naïve US military personnel. JAMA.

[9] Kim YJ, Bae JI, Ryoo SM, Kim WY (2019). Acute fulminant myocarditis following influenza vaccination requiring extracorporeal membrane oxygenation. Acute Crit Care.

[10] Goriely S., Goldman M. (2007). From tolerance to autoimmunity: is there a risk in early life vaccination?. J Comp Pathol.

[11] Kostoff R.N., Kanduc D., Porter A.L., Shoenfeld Y, Calina D (2020). Vaccine- and natural infection-induced mechanisms that could modulate vaccine safety. Toxicol Rep.

[12] Talotta R. (2021). Do COVID-19 RNA-based vaccines put at risk of immune-mediated diseases? In reply to “Potential antigenic cross-reactivity between SARS-CoV-2 and human tissue with a possible link to an increase in autoimmune diseases”. Clin Immunol.

[13] Abu Mouch S, Roguin A, Hellou E, Ishai A, Shoshan U (2021). Myocarditis following COVID-19 mRNA vaccination. Vaccine.

[14] Marshall M, Ferguson ID, Lewis P, Jaggi P, Gagliardo C (2021). Symptomatic Acute Myocarditis in Seven Adolescents Following Pfizer-BioNTech COVID-19 Vaccination. Pediatrics.

[15] Ammirati E, Cavalotti C, Milazzo A, Pedrotti P, Soriano F (2021). Temporal relation between second dose BNT162b2 mRNA Covid-19 vaccine and cardiac involvement in a patient with previous SARS-COV-2 infection. IJC Heart Vasc.

[16] Bautista Garcıa J, Peña Ortega P, Bonilla Fernández JA, Cárdenes León, Ramírez Burgos (2021). Acute myocarditis after administration of the BNT162b2 vaccine against COVID-19. Rev Esp Cardiol.

[17] Albert E, Aurigemma G, Saucedo J, Hoffman D, McClenathan B (2021). Myocarditis following COVID-19 vaccination. Radiol Case Rep.

[18] Watkins K, Griffin G, Septaric K, Simon EL (2021). Myocarditis after BNT162b2 vaccination in a healthy male. Am J Emerg Med.

[19] Kim HW, Jenista ER, Wendell DC, Azevedo CF, Campbell MJ (2021). Patients With Acute Myocarditis Following mRNA COVID-19 Vaccination. JAMA Cardiol.

[20] Montgomery J, Ryan M, Engler R, Hoffman D, McClenathan B (2021). Myocarditis Following Immunization With mRNA COVID-19 Vaccines in Members of the US Military. JAMA Cardiol.

[21] Gatti M, Carisio A, D’Angelo T, Darvizeh F, Dell’Aversana S (2020). Cardiovascular magnetic resonance in myocardial infarction with non-obstructive coronary arteries patients: A review. World J Cardiol.

[22] Patrignani A, Palmieri G, Ciampani N, Moretti V, Mariani A (2018). Under-reporting of adverse drug reactions, a problem that also involves medicines subject to additional monitoring. Preliminary data from a single-center experience on novel oral anticoagulants. G Ital Cardiol.

[23] Segec A, Slattery J, Morales DR, Januskiene J, Kurz X (2021). Does additional monitoring status increase the reporting of adverse drug reactions? An interrupted time series analysis of EudraVigilance data. Pharmacoepidemiol Drug Saf.

[24] Caforio ALP, Adler Y, Agostini C, Allanore Y, Anastasakis A (2017). Diagnosis and management of myocardial involvement in systemic immune-mediated diseases: a position statement of the European Society of Cardiology Working Group on Myocardial and Pericardial Disease. Eur Heart J.

[25] Karki R, Janga C, Deshmukh AJ (2020). Arrhythmias Associated with Inflammatory Cardiomyopathies. Curr Treat Options Cardiovasc Med.

[26] Lynge TH, Nielsen TS, Gregers Winkel B, Tfelt-Hansen J, Banner J (2019). Sudden cardiac death caused by myocarditis in persons aged 1-49 years: a nationwide study of 14 294 deaths in Denmark. Forensic Sci Res.

[27] Engler RJ, Nelson MR, Collins LC, Spooner C, Hemann BA (2015). A prospective study of the incidence of myocarditis/pericarditis and new onset cardiac symptoms following smallpox and influenza vaccination. PLoS One.

